# Exploring Spiritual Needs and Loneliness Among Acutely Hospitalized Patients with Chronic Illnesses: An Observational Study Across Three Waves of the COVID-19 Epidemic in Taiwan

**DOI:** 10.3390/medicina61040770

**Published:** 2025-04-21

**Authors:** Yu-Yin Kao, Shu-Wei Wang, Chen-Hsiang Lee

**Affiliations:** 1 Department of Nursing, Kaohsiung Chang Gung Memorial Hospital, Kaohsiung 833, Taiwan; yuyin0020@cgmh.org.tw; 2 Department of Nursing, Asia University, Taichung 413, Taiwan; shuwei@asia.edu.tw; 3 Department of Internal Medicine, Division of Infectious Diseases, Kaohsiung Chang Gung Memorial Hospital, College of Medicine, Chang Gung University, Kaohsiung 833, Taiwan; 4 Department of Internal Medicine, Division of Infectious Diseases, Chiayi Chang Gung Memorial Hospital, Chiayi 613, Taiwan

**Keywords:** loneliness, spiritual needs, social isolation, coronavirus disease 2019

## Abstract

*Background and Objectives*: Spirituality and loneliness are widely recognized as important aspects of holistic healthcare. This observational study was conducted among hospitalized patients with chronic illnesses in a medical ward during three waves of the epidemic in Taiwan, from April 2022 to March 2023, to examine changes in spiritual needs and loneliness. *Materials and Methods*: These waves were classified as the first wave (outbreak period, April 2022 to July 2022); second wave (mitigation period, August 2022 to November 2022); and third wave (December 2022 to March 2023). The Spiritual Needs Scale and Loneliness Scale were used to assess changes in spiritual needs and loneliness of the enrolled patients across the different waves of the epidemic. *Results*: We found that the spiritual needs of the enrolled patients were higher during the outbreak period (*F* = 9.847, *p* < 0.001) compared to the other periods. In addition, loneliness was higher during the conclusion period (*F* = 45.764, *p* < 0.001) compared to the other two periods. Age (*r* = 0.261, *p* < 0.001) and the Charlson comorbidity index (*r* = 0.193, *p* < 0.01) were significantly positively correlated with spiritual needs. Furthermore, the number of daily confirmed COVID-19 cases (*r* = −0.392, *p* < 0.001) was significantly negatively correlated with loneliness. *Conclusions*: Hospitalized patients with chronic illnesses experienced heightened spiritual needs during the COVID-19 outbreak, while loneliness increased as the epidemic waned. The study’s prospective observational design is a strength, but incorporating additional temporal measures between periods would have enhanced the findings.

## 1. Introduction

The COVID-19 pandemic has profoundly influenced societies globally, impacting various sectors such as health, employment, education, recreation, and the economy. During this interval, rigorous control strategies, particularly social distancing, were enacted across most nations to alleviate and stabilize COVID-19 infections [1,2]. These strategies, encompassing school and restaurant closures, restrictions on large assemblies, and travel limitations, culminated in increased prevalence rates of solitude and social seclusion in comparison to pre-pandemic levels [3]. Investigations indicated that up to 25% of individuals within the European Union frequently encountered feelings of loneliness and deteriorating psychological well-being during the pandemic [4]. Furthermore, extended quarantine protocols intensified the rising loneliness and social isolation as the pandemic continued [5,6]. Thus, the COVID-19 pandemic has been identified as a crucial determinant contributing to the rise in loneliness globally [6].

Loneliness is a subjective experience of disconnection, highlighting a disparity in social relationships [7]. Durmuş and Öztürk articulated the robust relationship between spirituality and loneliness, proposing that a more profound spiritual engagement could potentially mitigate social alienation [8]. However, differences in cultural contexts or personal spiritual rituals add to this complexity. During the pandemic, individuals, particularly the elderly and hospitalized, faced heightened loneliness despite seeking spiritual solace [4,9]. Spiritual well-being involves an individual’s capability to maintain resilience amid substantial life stressors. Research underscores spirituality’s vital role in emotional management during illness [10]. Investigations have shown a favorable relationship between spiritual wellness and the capacity for resilience. Individuals exhibiting elevated levels of spiritual well-being reveal enhanced coping mechanisms and a heightened ability to extract meaning and hope from their experiences [11,12]. Moreover, engagement in spiritual practices and adherence to beliefs throughout the pandemic have demonstrated a correlation with psychological alleviation and an enhancement in resilience [13,14]. Nonetheless, the insufficient expression of spiritual needs among hospitalized patients points to the necessity of integrating these considerations within healthcare to alleviate isolation’s emotional consequences.

In Taiwan, the government implemented stringent control measures early in the epidemic, enhancing infection control and visitor restrictions in healthcare settings, thereby limiting patient–family interactions. Nevertheless, Taiwan faced its inaugural local COVID-19 outbreak in April 2022, with daily cases exceeding 10,000 on April 28, 2022 [15]. During the lengthy period of two and a half years spent in quarantine, patients who were hospitalized faced significant emotional turmoil stemming from their social isolation, which obstructed their capacity to share their anxieties with loved ones and friends [16,17,18]. During this time frame, individuals exhibited intensified emotional reactions, such as panic, anger, anxiety, and worry, particularly in the early phases [19]. As isolation persisted, many individuals reported increased feelings of loneliness, helplessness, and depression. Among chronically ill patients hospitalized with acute conditions, the stress from health concerns, rigorous treatment protocols, and familial worries exacerbated both emotional and physical difficulties [20,21]. Previous studies have elucidated restrictive measures implemented by various countries principally designed to reduce social interactions and mobility amidst the pandemic. These interventions concomitantly resulted in an escalation of feelings of loneliness within the community [22]. In contrast, when considering the ramifications of social isolation on hospitalized individuals suffering from chronic illnesses, the impact may be considerably more pronounced in comparison to that on the general population [20,21]. To facilitate efficacious support, a comprehensive understanding of the spiritual needs and experiences of loneliness among acutely hospitalized patients with chronic illnesses during the COVID-19 pandemic is required, and this is frequently where a significant gap exists.

After the pandemic’s peak, many nations relaxed infection controls. However, Taiwan continued rigorous visitor restrictions for hospitalized patients throughout the entire duration [23]. This study investigated the effects of these restrictions on the spiritual needs and loneliness of acute hospitalized patients with chronic diseases during three epidemic waves in Taiwan from April 2022 to March 2023. By addressing the psychological and spiritual requirements of these patients, the research aimed to inform healthcare professionals in alleviating loneliness, fulfilling spiritual needs, and improving care strategies for future.

## 2. Materials and Methods

### 2.1. Study Design and Setting

This observational study was undertaken in a southern Taiwan medical center’s internal medicine ward. The research time frame extended from April 2022 to March 2023, covering three distinct COVID-19 epidemic waves, each approximately four months long. The first wave (April to July 2022) represented the outbreak phase, with daily confirmed cases peaking at over 90,000. The second wave (July to November 2022) indicated a mitigation phase, with daily confirmed cases averaging around 40,000. The third wave (December 2022 to March 2023) signified the conclusion phase, with daily confirmed cases averaging around 20,000. This phase was attributed to high vaccination rates (93% for the first dose, 88% for the second dose), the relaxation of mask mandates from December 2022, and increased public awareness of COVID-19 and effective protective strategies [24,25]. Each of the three waves underwent analysis utilizing a consistent data presentation methodology to ensure comparability and interpretation uniformity.

### 2.2. Participant Recruitment and Data Collection

The principal investigator elucidated the study’s aims and methodologies to prospective subjects prior to their enrollment. Following the acquisition of informed consent, participants filled out structured questionnaires independently upon admission. The assessment comprised three standardized questionnaires: a demographic survey, the Spiritual Needs Scale, and the Loneliness Scale. This study was reviewed and approved by the Institutional Review Board (IRB No.: 202200244B0C502). All participating patients engaged voluntarily and were informed of their right to withdraw at any point without consequences. The inclusion criteria for participants are as follows: (1) having one or more chronic illnesses, including hypertension, hyperlipidemia, arthritis, diabetes, chronic kidney disease, heart failure, chronic liver disease, or cerebrovascular disease; (2) being between 20 and 79 years old; and (3) having clear consciousness and the ability to communicate in Mandarin or Taiwanese. The exclusion criteria encompassed individuals with mental health disorders such as schizophrenia, bipolar disorder, or major depressive disorder. Each patient was enrolled only once, when first admitted during the study period, and was not recruited again. The sample size was determined utilizing G-Power 3.1 software, incorporating a significance level (α) of 0.05, an anticipated effect size of 0.5, and a statistical power of 0.95. The minimum sample size required was 252, evenly allocated across three groups (84 participants each).

### 2.3. Demographic Data and Psychosocial Measures

Data on gender, age, marital status, education level, occupation, religious beliefs, and chronic disease status were collected. The chronic disease status of participants was evaluated using Charlson comorbidity index (CCI) [26].

The Spiritual Needs Questionnaire (SpNQ-Ch-27) was adapted into Chinese by Zhao et al., based on Büssing’s original scale [27,28]. This instrument consists of 27 items organized into six dimensions. Inner Peace Needs pertain to emotional stability and safety. Actively Giving Needs reflect the desire to support others. Religious Needs (Praying) involve a connection with a higher power through prayer. Religious Needs (Sources) focus on seeking guidance from religious texts or leaders. Existential Needs (Reflection) relate to the exploration of life’s purpose and value. Existential Needs (Release) concern achieving psychological relief or alleviating worries. Responses are measured on a 4-point Likert scale, resulting in total scores from 27 to 108, with higher scores indicating heightened spiritual needs [28].

The Loneliness Scale, derived from De Jong Gierveld and Kamphuis, contains nine items [29]. It features two subscales: Social Loneliness (5 items) and Emotional Loneliness (4 items). Participants respond with “yes”, “no”, or “neutral”, yielding scores from 0 to 9, with higher scores reflecting heightened loneliness [29,30].

### 2.4. Statistical Analysis

Quantitative data were analyzed using SPSS version 24 (IBM Inc., Armonk, NY, USA). Demographic characteristics were assessed by frequency and percentage. Continuous data were presented as mean and standard deviation, and ANOVA was performed to examine any differences. Differences in spiritual needs and loneliness across the three waves were assessed using the chi-squared test, ANOVA, and Fisher’s exact test. All tests were two-tailed with a significance level of *p* < 0.05.

## 3. Results

### 3.1. Demographic Differences Across the Three Waves

A cumulative total of 295 individuals (97, 99, and 99 across the three distinct waves, respectively) were recruited for participation. The findings conform to the fundamental criteria for a sufficient sample size. The demographic characteristics of participants across the three waves revealed significant differences in elderly patients (aged ≥65 years) (*p* = 0.017) and according to employment status (*p* = 0.002) and religious beliefs (*p* = 0.026). No significant variations were observed regarding gender, marital status, educational attainment, acute illness classification, and chronic illness status among participants across the three waves (Table 1).

In the initial wave (outbreak period), a larger segment of participants was elderly (58.8%), in contrast to 38.3% and 48.5% observed in the second (mitigation period) and third (conclusion period) waves, respectively. Furthermore, the employment rate reached its apex during the second wave (43.4%) before declining in the subsequent third wave, suggesting potential socioeconomic ramifications over time. The percentage of participants identifying with religious beliefs exhibited fluctuations across the waves, marked by a significant reduction during the second wave (71.7%) followed by a resurgence in the third wave (80.8%). This variability underscores the conceivable function of religion as a coping mechanism throughout the three waves.

### 3.2. Fluctuations in Spiritual Needs

**Figure 1:**
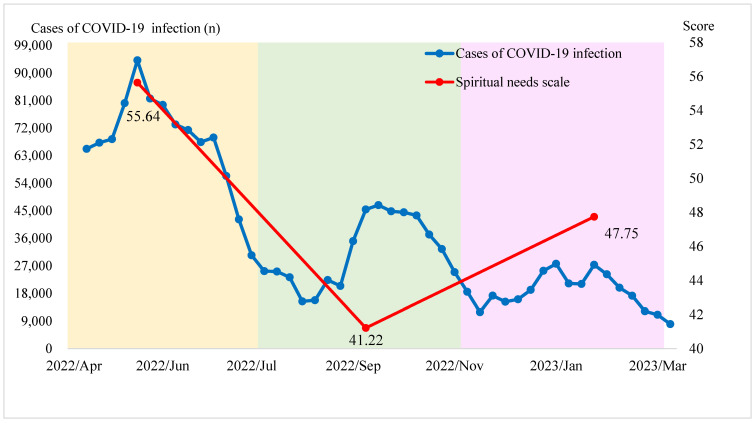
Cases of COVID-19 infection and Spiritual Needs Scale of enrolled patients in the three waves of the COVID-19 epidemic in Taiwan. Compared with the spiritual needs of patients in the second wave (T2) and the third wave (T3), those of the patients were significantly higher in the first wave (T1).

Spiritual needs were significantly higher in the first wave compared to the second and third waves (*F* = 9.847, *p* < 0.001) (Table 2, Figure 1). Subcategories such as Inner Peace Needs (*F* = 9.754, *p* < 0.001) and Religious Needs—Praying (*F* = 9.231, *p* < 0.001) were particularly elevated during the outbreak period, reflecting the heightened psychological distress and uncertainty faced by participants. Spiritual needs positively correlated with age (*r* = 0.261, *p* < 0.001) and the Charlson comorbidity index (*r* = 0.193, *p* < 0.01) (Table 3).

### 3.3. Evolution of Loneliness Across Waves

**Figure 2:**
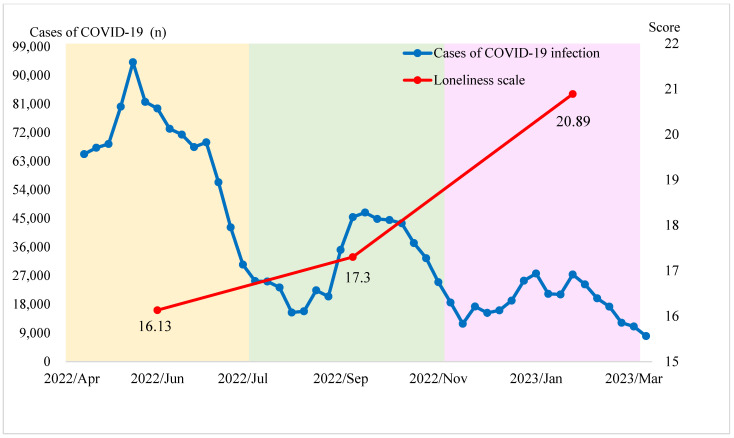
Cases of COVID-19 infection and Loneliness Scale of enrolled patients in the three waves of the COVID-19 epidemic in Taiwan. Compared with the Loneliness Scale of patients in the first wave (T1) and the second wave (T2), those of the patients were significantly higher in the third wave (T3).

Loneliness scores significantly increased during the third wave, with the highest mean score of 20.89 ± 1.91 compared to 16.13 ± 4.64 and 17.31 ± 3.81 in the first and second waves, respectively (*F* = 45.764, *p* < 0.001) (Table 2, Figure 2). The social loneliness subscale was higher than the emotional loneliness subscale in the third wave, indicating a stronger impact of disrupted social connections rather than individual emotional distress. Remarkably, loneliness scores negatively correlated with the number of COVID-19 cases (*r* = −0.392, *p* < 0.001), suggesting that reduced case numbers during the conclusion period may have led to diminished collective solidarity and increased perceived isolation (Table 3).

### 3.4. Correlations Between Epidemic Dynamics and Psychological Indicators

A considerable positive association was identified between the number of COVID-19 cases and participants’ Existential Needs—Release (*r* = 0.132, *p* < 0.05), further emphasizing the heightened psychological and spiritual distress during periods of high infection rates (Table 3). Conversely, the number of COVID-19 cases exhibited a negative correlation with loneliness scores (*r* = −0.392, *p* < 0.001) (Table 3), underscoring a multifaceted interaction between external epidemic influences and individual psychological conditions.

## 4. Discussion

In this study, we categorized the COVID-19 epidemic in Taiwan into three waves following the methodology established by Chen et al. [15]. The analysis illuminated the government’s stringent control measures during the initial wave from April 2022 to July 2022 in response to the evolving epidemiological landscape. Following a decline in confirmed cases, outdoor and school mask mandates were relaxed during the second wave from July 2022 to November 2022. The third wave, spanning from December 2022 to March 2023, witnessed a resurgence in confirmed cases. Given the high vaccination rate, the government has progressively shortened quarantine duration and promoted self-health management. This strategy suggests effective pandemic control, supporting the classification of the epidemic into three waves as a valid approach.

Tailored programs offering emotional and spiritual support can mitigate the adverse effects of global health crises on vulnerable groups. Research in areas heavily impacted by COVID-19 has revealed increased reliance on spirituality among community members facing social isolation [31]. Likewise, surveys across various cultures have indicated that many respondents viewed religious beliefs as vital resources during the pandemic [32]. Conversely, hospitalized patients with chronic conditions demonstrated heightened spiritual needs, influenced by uncertainty, fear of infection, and isolation from family due to strict visitation rules [17,33]. These disruptions significantly affected daily life and healthcare, leading to psychological responses like anxiety, depression, and loneliness. These observations are consistent with a systematic review by Desmet et al., which underscored the necessity of addressing spiritual needs, especially in older hospitalized patients [34]. Notably, the inability to maintain familial connections exacerbated feelings of vulnerability, motivating hospitalized individuals to seek comfort and meaning through spirituality and religiosity [32,35].

Spiritual needs are closely related to emotional and psychological conditions, often leading to increased spiritual pursuits during crises [36]. Addressing spiritual needs is crucial in reducing emotional and existential suffering. Online communication and interventions significantly alleviated these issues, especially amid limited physical interactions [32,37]. Participants reported elevated loneliness during the epidemic’s third wave compared to earlier stages. Most hospitals adopted remote rounds to replace traditional bedside rounds after the outbreak of the pandemic. Healthcare workers wore isolation suits and goggles, making communication with the patients more challenging. Collectively, these factors intensified loneliness among patients with chronic illnesses, particularly during the pandemic’s initial phase [38]. Few studies have longitudinally examined loneliness among hospitalized patients post the COVID-19 pandemic [39]. Our results indicated an increase in social loneliness among patients, particularly during the epidemic’s third wave (Figure 2). This rise may originate from authorized social engagements that marginalized chronically ill hospitalized patients, exacerbating their social loneliness. Participants reported heightened spiritual needs during the pandemic’s peak compared to other periods. In contrast, they experienced lower loneliness and social isolation levels during the pandemic’s outbreak relative to other times. Recent research underscores the importance of addressing spiritual needs in healthcare settings. Evidence suggests that meeting the spiritual needs of individuals with chronic illnesses can alleviate crisis-related adverse effects [40]. Additional studies indicate that spirituality and religious beliefs can confer health protection [41]. Systematic reviews have shown that increased spiritual and religious demands, coupled with a proactive life approach, may alleviate loneliness and fear linked to social isolation during the COVID-19 pandemic [11,42]. Furthermore, individuals often turn to spiritual needs and beliefs to foster inner peace and happiness [35,43]. This highlights the critical role of spiritual needs in mental health and indicates that spirituality may facilitate coping with adversity.

In this study, older age and an increased Charlson comorbidity index correlated with elevated spiritual needs and loneliness. This aligns with prior research indicating that older age and chronic illness prevalence are linked to heightened spiritual needs in Denmark [43]. Additional studies have demonstrated a relationship between older age and increased loneliness. The findings suggest that reduced physical activity in chronic illness patients may limit social engagement, leading to greater loneliness [20]. Furthermore, older adults with chronic illnesses exhibited pronounced emotional loneliness compared to social loneliness. Conversely, younger individuals tend to prioritize social needs, notably during COVID-19 restrictions, exacerbating social loneliness. One study indicated that younger populations in Europe experienced intensified loneliness due to social isolation amid the pandemic [4]. The authors attributed this primarily to constraints on economic and material necessities during lockdowns. The study noted that the fulfilment of material needs was associated with diminished loneliness levels. In Taiwan, the provision of meals and financial support during the pandemic’s onset resulted in lower loneliness rates among patients [28]. However, as confirmed cases rose and public space restrictions eased, hospitals in Taiwan maintained visitor limitations. Consequently, patients reported increased social loneliness, wishing for emotional support and companionship during hospitalization as public isolation measures relaxed. This aligns with numerous studies highlighting a rise in loneliness and social isolation over time due to the COVID-19 pandemic’s effects [2,3,6]. These psychological challenges accumulate progressively from social isolation, resulting in heightened loneliness.

### Limitations

Conducting three separate cross-sectional studies over different time periods with small sample sizes from a single tertiary hospital might not be sufficient at all for the objectives of this project. Future studies should implement multicenter surveys to enhance sample diversity and generalizability. Moreover, research with prolonged follow-up is essential to comprehensively understand the physiological, psychological, and spiritual transformations of hospitalized patients in social isolation contexts. In addition, demographic variations such as age, employment status, and religious affiliation throughout diverse study waves may result in potential biases in the analysis of the data. Nevertheless, one of the strengths of the present study is its prospective nature, characterized by observational methodologies; however, it would have been advantageous to integrate additional temporal assessment measures between the three established time periods. The provision of spiritual care within healthcare facilities has highlighted the ad hoc nature of spiritual and pastoral care delivery, indicating a pressing need to address this imbalance [44].

## 5. Conclusions

Our research indicates that hospitalized chronic illness patients experienced heightened spiritual needs and isolation during the COVID-19 epidemic’s later stages. The ongoing infection control measures, including visitor restrictions, likely exacerbated feelings of loneliness in the epidemic’s concluding phase. Hence, addressing the spiritual requirements of these patients is crucial in mitigating isolation and enhancing their coping strategies.

## Figures and Tables

**Table 1:** The demographics of enrolled patients in the three waves of the COVID-19 epidemic in Taiwan.

Variables	T1 (*n* = 97) April–July 2022	T2 (*n* = 99) August–November 2022	T3 (*n* = 99) December 2022–March 2023	*p*
	*n* (%)	*n* (%)	*n* (%)	
Age (years)				
Range	24–79	20–79	20–79	0.107
≥ 65	57 (58.8)	38 (38.3)	48 (48.5)	0.017
Gender				0.965
Male	55 (56.7)	58 (58.6)	57 (57.6)	
Female	42 (43.3)	41 (41.4)	42 (42.4)	
Marital status				0.380
Single/widowed	23 (23.7)	32 (32.3)	30 (30.3)	
Married/partnered	74 (76.3)	67 (67.7)	69 (69.7)	
Educational level				0.234
Elementary school and below	36 (37.1)	25 (25.2)	32 (32.3)	
Junior and senior high school	42 (43.3)	50 (50.5)	39 (39.4)	
College degree or above	19 (19.6)	24 (24.3)	28 (28.3)	
Employment status				0.002
Employed	25 (25.8)	43 (43.4)	38 (38.4)	
Religion				0.026
Yes	78 (80.4)	71 (71.7)	80 (80.8)	
Acute illness				0.062
Infectious diseases	53 (54.6)	54 (54.5)	72 (72.7)	
Gastrointestinal diseases	17 (17.5)	21 (21.2)	12 (12.1)	
Malignancy	26 (26.8)	22 (22.2)	14 (14.1)	
Cardiovascular diseases	1 (1.1)	2 (2.1)	1 (1.1)	
Charlson comorbidity index (score)				0.494
0–2	20 (20.6)	32 (32.3)	26 (26.3)	
3–5	31 (32.0)	30 (30.3)	29 (29.3)	
>6	46 (47.4)	37 (37.4)	42 (44.4)	

The first wave (T1), the second wave (T2), and the third wave (T3).

**Table 2:** Cases of COVID-19 infection and comorbidity, spiritual needs, and loneliness of the enrolled patients in the three waves of the COVID-19 epidemic in Taiwan.

Variables	T1 (*n* = 97) 2022/Apr–2022/Jul	T2 (*n* = 99) 2022/Aug–2022/Nov	T3 (*n* = 99) 2022/Dec–2023/Mar	*F*	*p*
Number of COVID-19 cases (*n*)	33,056.12 ± 12,204.19	26,730.77 ± 10,873.39	4746.35 ± 4265.72	229.275	<0.001
Charlson comorbidity index (score)	5.14 ± 3.06	4.67 ± 3.44	5.01 ± 3.59	0.506	0.603
Spiritual Needs (score)	55.64 ± 19.00	41.22 ± 24.29	47.76 ± 24.26	9.847	<0.001
Inner Peace Needs	15.78 ± 4.88	12.03 ± 6.89	13.87 ± 5.89	9.754	<0.001
Actively Giving Needs	14.01 ± 5.89	10.83 ± 6.91	11.82 ± 7.13	5.827	0.003
Religious Needs—Praying	5.46 ± 3.16	3.49 ± 3.23	4.67 ± 3.29	9.231	<0.001
Religious Needs—Sources	7.01 ± 3.86	5.17 ± 4.32	6.31 ± 4.24	4.909	0.008
Existential Needs—Reflection	10.21 ± 2.54	7.29 ± 4.15	8.02 ± 3.21	19.772	<0.001
Existential Needs—Release	3.16 ± 2.12	2.31 ± 2.26	3.07 ± 2.18	4.485	0.012
Loneliness (score)	16.13 ± 4.64	17.31 ± 3.81	20.89 ± 1.91	45.764	<0.001
Social loneliness	8.62 ± 1.93	9.91 ± 2.56	9.61 ± 1.90	9.621	<0.001
Emotional loneliness	9.80 ± 0.85	8.83 ± 1.44	8.71 ± 0.98	28.199	<0.001

The first wave (T1), the second wave (T2), and the third wave (T3).

**Table 3:** Variable correlations of the enrolled patients.

Parameter	Cases of COVID-19 Infection (*n*)	Charlson Comorbidity Index	Spiritual Needs	Existential Needs—Release	Existential Needs—Reflection	Inner Peace Needs	Actively Giving Needs	Religious Needs—Praying	Religious Needs—Sources	Loneliness	Social Loneliness	Emotional Loneliness
Age (year)	0.100	0.582 **	0.261 ***	0.258 ***	0.191 **	0.225 ***	0.227 ***	0.259 ***	0.263 ***	0.007	−0.271 ***	0.171 **
Cases of COVID-19 infection (*n*)	1	0.082	−0.025	0.132 *	−0.113	−0.041	−0.027	−0.031	−0.056	−0.392 ***	−0.121 *	0.258 ***
Charlson comorbidity index (score)		1	0.193 **	0.198 **	0.136 *	0.191 **	0.154 **	0.164 **	0.200 **	−0.007	−0.182 **	0.141 *
Spiritual Needs (score)			1	0.753 ***	0.878 ***	0.891 ***	0.941 ***	0.898 ***	0.924 ***	−0.041	−0.225 ***	0.200 **
Existential Needs—Release				1	0.513 ***	0.797 ***	0.566 ***	0.541 ***	0.556 ***	−0.109	−0.281 ***	0.395 ***
Existential Needs—Reflection					1	0.706 ***	0.847 ***	0.812 ***	0.872 ***	0.000	−0.184 **	0.094
Inner Peace Needs						1	0.747 ***	0.666 ***	0.709 ***	−0.017	−0.259 ***	0.212 ***
Actively Giving Needs							1	0.883 ***	0.895 ***	−0.052	−0.162 **	0.106
Religious Needs—Praying								1	0.899 ***	−0.028	−0.204 ***	0.169 **
Religious Needs—Sources									1	−0.022	−0.143 *	0.125 *
Loneliness (score)										1	0.340 ***	−0.134 *
Social loneliness											1	−0.311 ***
Emotional loneliness												1

* *p* < 0.05, ** *p* < 0.01, *** *p* < 0.001; data analysis by Pearson correlation

## Data Availability

The data associated with the paper are not publicly available but are available from the corresponding author on reasonable request.
